# Functional Laryngeal Assessment in Patients with Tracheostomy Following COVID-19 a Prospective Cohort Study

**DOI:** 10.1007/s00455-022-10496-4

**Published:** 2022-07-16

**Authors:** C. Dawson, P. Nankivell, J. P. Pracy, R. Capewell, M. Wood, J. Weblin, D. Parekh, J. Patel, S. A. Skoretz, N. Sharma

**Affiliations:** 1 Department of Therapy Services, Queen Elizabeth Hospital Birmingham NHSFT, Birmingham, UK; 2 University of Birmingham Institute of Clinical Sciences, Birmingham, UK; 3 School of Audiology and Speech Sciences, University of British Columbia, Vancouver, Canada; 4 Institute of Cancer and Genomic Sciences, University of Birmingham, Birmingham, UK; 5 Department of Otolaryngology, Queen Elizabeth Hospital, Birmingham, UK; 6 Centre for Translational Inflammation and Fibrosis Research, School of Clinical and Experimental Medicine, University of Birmingham, Birmingham, UK; 7 Department of Anaesthetics and Critical Care, Queen Elizabeth Hospital Birmingham, Birmingham, UK; 8 Department of Critical Care Medicine, University of Alberta, Edmonton, Canada; 9 Centre for Heart Lung Innovation, St. Paul’s Hospital, Providence Health Care, Vancouver, Canada

**Keywords:** COVID-19, Decannulation, Extubation, Intubation, Larynx, Tracheostomy, Rehabilitation

## Abstract

To explore laryngeal function of tracheostomised patients with COVID-19 in the acute phase, to identify ways teams may facilitate and expedite tracheostomy weaning and rehabilitation of upper airway function. Consecutive tracheostomised patients underwent laryngeal examination during mechanical ventilation weaning. Primary outcomes included prevalence of upper aerodigestive oedema and airway protection during swallow, tracheostomy duration, ICU frailty scores, and oral intake type. Analyses included bivariate associations and exploratory multivariable regressions. 48 consecutive patients who underwent tracheostomy insertion as part of their respiratory wean following invasive ventilation in a single UK tertiary hospital were included. 21 (43.8%) had impaired airway protection on swallow (PAS ≥ 3) with 32 (66.7%) having marked airway oedema in at least one laryngeal area. Impaired airway protection was associated with longer total artificial airway duration (*p* = 0.008), longer tracheostomy tube duration (*p* = 0.007), multiple intubations (*p* = 0.006) and was associated with persistent ICU acquired weakness at ICU discharge (*p* = 0.03). Impaired airway protection was also an independent predictor for longer tracheostomy tube duration (*p* = 0.02, Beta 0.38, 95% CI 2.36 to 27.16). The majority of our study patients presented with complex laryngeal findings which were associated with impaired airway protection. We suggest a proactive standardized scoring and review protocol to manage this complex group of patients in order to maximize health outcomes and ICU resources. Early laryngeal assessment may facilitate weaning from invasive mechanical ventilation and liberation from tracheostomy, as well as practical and objective risk stratification for patients regarding decannulation and feeding.

## Introduction

Severe acute respiratory syndrome coronavirus 2 (SARS-CoV-2) results in a high incidence of respiratory distress. Between 1/9/20–10/5/21, 7644 individuals in the UK received invasive ventilation within 24 h of admission to hospital [1]. These patients often receive prolonged mechanical ventilation, prone positioning, deep therapeutic sedation, and the use of muscle relaxants and tracheostomies [2]. While tracheostomy tubes are often necessary for survival, the iatrogenic effects of these and other artificial airways on the larynx are still emerging.

Tracheostomy decannulation timing and approaches are numerous [3]. A key question for teams managing patients with tracheostomy tubes relates to timing of safe decannulation. This important rehabilitation consideration facilitates independence, improves communication, reduces dependence on therapeutic interventions (e.g., tube feeding), and improves health outcomes [4]. In most acute care facilities, decision-making for tracheostomy decannulation is made using multi-disciplinary clinical care pathways. Institution specific protocols tend to govern this decision-making rather than national guidelines [3]. During the COVID-19 pandemic, decannulation decisions have been challenging due to healthcare system burden as well as the complex clinical presentation; specifically, delirium management [5], gross inflammatory process and cytokine storms [6], hypoxemia [7], coagulation disorders [8], swallowing impairment or dysphagia [9], mobility and fatigue [10], and upper airway compromise [11]. In order to explore ways to facilitate tracheostomy weaning and expedite rehabilitation of upper airway function, we present our quality assurance audit following laryngeal assessment of patients with severe COVID-19 who underwent tracheostomy tube insertion.

## Methods

Prospective data collection was used to gather information on acute laryngeal anatomy and physiology following invasive ventilation and tracheostomy tube placement for treatment of COVID-19. Nasendoscopic examination before decannulation is standard practice in our institution. There is no control group in this study, as the evaluation was undertaken during the second peak of Covid-19 in the UK, where there was no available comparable group due to the intense resource utilisation for COVID-19 patients. We seek to describe this specific pathophysiology, to inform other ENT, intensive care and Speech and Language Therapy teams and to improve outcomes for this complex group of patients. Future prospective work is underway to facilitate comparison between case–control with appropriate methodology and study design. This work uses data provided by patients and collected by the NHS as part of their care and support at the Queen Elizabeth Hospital Birmingham NHS Foundation Trust. It has been approved by University Hospitals Birmingham NHS Foundation Trust, Clinical Audit Registration & Management System and the COVID-19 research facilitation group under application reference CARMS-17155.

## Setting

From January 1 to April 28, 2021, we included all consecutive ICU patients considered for tracheostomy weaning at the Queen Elizabeth Hospital Birmingham. Included were those with severe respiratory failure secondary to SARS-CoV-2 with positivity confirmed by real-time polymerase chain reaction testing (nasopharyngeal swabs) or non-directed bronchial lavage/aspirate. Once the patient was able to tolerate oxygen delivery via tracheostomy mask (without invasive ventilation), they were eligible for a fiberoptic nasendoscopy.

### Tracheostomy Multi-Disciplinary Team (MDT)

We have described percutaneous tracheostomy tube insertion methods and operational aspects of our MDT previously [2]. In brief, the majority of tracheostomies were percutaneous, undertaken by a surgical team with therapeutic management led by the Speech and Language Therapists (SLT). Other MDT personnel included: ENT, physiotherapists, altered airway nurses, education leads, respiratory physicians, intensivists, and ward nursing staff.

### Laryngeal Assessment

An ENT surgeon and SLT completed the laryngeal assessment via nasendoscopy when the patient tolerated oxygen supplementation via a tracheostomy mask without invasive ventilation. Where patients were sufficiently alert, a swallow assessment was completed with and diet fluid recommendations. The examination was recorded on the AMBU disposable scope system (Ambu® aScope™ 4 RhinoLaryngo Slim). We used a predefined proforma, including laryngeal and pharyngeal motor and sensory assessment and standardised oedema and airway protection scoring during swallowing. The decision to decannulate was made by the treating team (intensive care or respiratory) using their usual clinical parameters. Data collection for audit purposes ceased after decannulation.

### Scales, Scoring, and Statistical Analyses

The revised Patterson oedema scale [12] and Penetration Aspiration scale (PAS)[13] were used. The revised Patterson Oedema scale is a standardized scoring method to rate upper airway oedema and the PAS is a validated tool which describes airway protection impairment. Raters for all airway scales were SLT and ENT surgeons with expertise in aerodigestive tract anatomy and physiology. Following institutional reliability training, two raters scored each laryngeal assessment with disagreements resolved by consensus. Scores of each component were recorded according to two regions based on anatomical location: region 1 (glottis) included the true vocal fold, false vocal fold, arytenoid and aryepiglottic components; and region 2 (supraglottis) included epiglottis, pharyngo-epiglottic folds, vallecula, and pyriform sinus components.

Presence of Intensive Care acquired weakness (ICUAW) was assessed by a physiotherapist at ICU discharge using Medical Research Council (MRC) sum score [14]. A validated tool within ICU, the MRC describes muscle power of each limb on an oxford scale from 0 (total paralysis) to 5 (normal power). ICUAW was defined as either ‘significant’ (MRC ≤ 48/60) or severe (MRC ≤ 36/60), with normal being an MRC > 48 [14]. Functional status was also assessed at ICU discharge using the Manchester Mobility Scale (MMS), a seven-point mobility scale validated for assessing mobility levels within ICU [15].

For analyses, we dichotomized PAS describing airway protection as ‘normal’ (PAS scores of either 1 or 2) or ‘impaired’ (PAS scores of 3 and above). All continuous variables were summarized according to mean with standard deviation (SD) and median with interquartile range (IQR) depending on whether data were normally distributed, and ordinal/categorical data summarized according to frequency counts. Following stratification of the sample according to normal or impaired PAS, bivariate comparisons were conducted using Mann–Whitney or unpaired 2-sided *t*-tests for continuous variables and Pearson’s χ^2^ test, likelihood ratio or Fisher’s exact test for proportions as appropriate. Post-hoc, we conducted two exploratory main effect regression analyses for two study outcomes: a logistic backward stepwise regression [16] to explore predictors for abnormal airway protection and a multiple linear regression to explore predictors for prolonged tracheostomy tube dependency. For these purposes, the following variables were defined as: ICU acquired weakness (yes [severe and significant]/no [normal]), abnormal false vocal fold oedema (moderate and severe), artificial airway duration (intubation + tracheostomy tube duration), and impaired airway protection (PAS ≥ 3). Given the exploratory nature of these regressions and the novel patient population, the five predictor variables which were chosen for our models were informed by our bivariate comparisons and clinical relevance [17]. Significance for all statistical tests was *p* < 0.05.

## Results

### Patient Characteristics

Of 146 consecutive patients, 48 met inclusion criteria during our study period. Mean age (SD) was 56.7 (10.7) years and 31 (64.6%) were male. On admission, the majority (27, 56.3%) had a Charlson comorbidity Index of 3 or above and 9 (18.8%) were living with mild pre-existing frailty before admission. Forty-one (85.4%) were without underlying chronic respiratory diseases (i.e., asthma and/or Chronic Obstructive Pulmonary Disease). Other demographics and baseline characteristics are available in Table 1.

**Table 1:** Demographics, baseline, respiratory and physical characteristics across the sample

Variable*	All patients (*N* = 48)	Airway protection		*p-*value
		Normal (*n* = 26)	Impaired (*n* = 21)	
Demographics and anthropometrics				
*Age*^a^				
Mean (SD)	56.7 (10.7)	54.0 (11.4)	60.0 (9.3)	0.06^1^
Median (IQR)	57.0 (14.0)	55.0 (14.0)	60.0 (13.0)	
*BMI*				
Mean (SD)	31.4 (7.7)	33.4 (9.6)	29.14 (3.3)	0.04^1^
Median (IQR)	29.0 (9.0)	30.5 (12.0)	29.0 (6.0)	
*Ethnicity *(%)				
White	22 (45.8)	11 (42.3)	11 (52.4)	0.06^4^
Asian	18 (37.5)	13 (50.0)	5 (23.8)	
Black	5 (10.4)	2.0 (7.7)	2 (9.5)	
Other	3 (6.3)	0 (0)	3 (14.3)	
*Sex *(%)				
Male	31 (64.6)	16 (61.5)	15 (71.4)	0.55^3^
Comorbid conditions				
*Asthma *(%)	7 (14.6)	7 (26.9)	0 (0)	0.01^3^
*Charlson comorbidity score *(%)				
0	2 (4.2)	1 (3.8)	1 (4.8)	0.07^4^
1	9 (18.8)	5 (19.2)	3 (14.3)	
2	10 (20.8)	7 (26.9)	3 (14.3)	
3	20 (41.7)	9 (34.6)	11 (52.4)	
≥ 4	7 (14.6)	4 (15.4)	3 (14.3)	
*Clinical Frailty Score *(%)^b^				
Very fit	10 (20.8)	5 (19.2)	4 (19.0)	0.51^4^
Fit	11 (22.9)	8 (30.8)	3 (14.3)	
Managing well	17 (35.4)	8 (30.8)	9 (42.9)	
Living with very mild frailty	9 (18.8)	4 (15.4)	5 (23.8)	
*COPD* (%)	1 (2.1)	0 (0)	1 (4.8)	0.45^3^
*DM* (%)	16 (33.3)	11 (42.3)	5 (23.8)	0.25^3^
*HTN* (%)	20 (41.7)	9 (34.6)	11 (52.4)	0.23^3^

*BMI* Body Mass Index, *COPD* Chronic obstructive pulmonary disease, *DM* Diabetes Mellitus, *HTN* hypertension

*Presented as n (%) unless otherwise indicated

^a^Reported in years

^b^Missing: No response 1 (2.1%) 1 normal airway missing

^1^ T-test

^2^Chi-Squared

^3^Fishers

^4^Likelihood Ratio

### Respiratory, Physical, and Airway Protection Characteristics

Respiratory and physical characteristics were reported/measured during the patients’ stay in the ICU and upon ICU discharge and transfer to the ward (Table 2). Across the sample, mean (SD) days with an artificial airway (endotracheal and tracheostomy tubes) were 49.7 (22.8) days. Duration of endotracheal or tracheostomy tubes separately were mean (SD) 17.9 (9.8) and 28.3 (17.6) days, respectively. Eleven patients (22.9%) required multiple intubations. The majority (44, 91.7%) required proning and paralysis during ventilation support with a mean (SD) of 23.9 (10.3) days of sedation for all patients. Following tracheostomy tube placement, 26 patients (54.2%) underwent Continuous Positive Airway Pressure (CPAP) trials during weaning. Nineteen (40%) patients were decannulated without requirement for tracheostomy tube downsizing. The remaining underwent tracheostomy downsizing after 10.4 (13.2) days. All patients were assessed for critical care weakness (based on MRC). Severe/very severe weakness was measured in 44 (91.7%) patients at the first rehabilitation session.

**Table 2:** Respiratory and physical characteristics across the sample

Variable*	All patients (*N* = 48)	Airway protection		*p-*value
		Normal (*n* = 26)	Impaired (*n* = 21)	
Respiratory				
*Total Days with Artificial Airway*				
Mean (SD)	49.7 (22.8)	46.5 (17.8)	54.2 (27.0)	0.008^1^
Median (IQR)	45.5 (25.8)	44.0 (26.0)	53.0 (31.3)	
*No. Undergoing Paralysis *(%)	44.0 (91.7)	23.0 (88.5)	20.0 (95.2)	1.00^3^
*Paralysis Days*				
Mean (SD)	17.8 (10.2)	17.4 (7.8)	19.3 (11.9)	0.14^1^
Median (IQR)	17.5 (17.0)	17.0 (14.0)	18.0 (21.0)	
*Proned *(%)	44.0 (91.7)	25.0 (96.2)	18.0 (85.7)	0.31^3^
*Sedation Days*				
Mean (SD)	23.9 (10.3)	22.8 (7.1)	25.8 (12.6)	0.07^1^
Median (IQR)	25.0 (17.0)	25.0 (12.0)	27.0 (23.0)	
*Days Intubated*				
Mean (SD)	17.9 (9.8)	16.9 (7.4)	19.6 (12.2)	0.38^1^
Median (IQR)	17.0 (14.0)	17.0 (12.0)	20.0 (23.0)	
*No. of Intubations *(%)				
1	35 (72.9)	24 (92.3)	10 (47.6)	0.006^4^
2	10 (20.8)	2 (7.7)	8 (38.1)	
3	1 (2.1)	0 (0.0)	1 (4.8)	
*Days with TT *(%)				
Mean (SD)	28.3 (17.6)	25.5 (14.7)	31.6 (20.6)	0.007^1^
Median (IQR)	24.5 (12.0)	24.0 (13.0)	25.0 (18.0)	
*CPAP Trial *(%)	26.0 (54.2)	15.0 (57.7)	10.0 (47.6)	0.56^3^
*Days to Downsize*(%)				
Mean (SD)	10.4 (13.2)	10.0 (7.9)	11.6 (17.3)	0.95^1^
Median (IQR)	6.0 (8.8)	6.0 (8.0)	5.0 (11.5)	
Physical				
*Critical Care Acquired Weakness* (%)**				
First Rehabilitation Session				
Severe	44 (91.7)	23 (88.5)	20 (95.2)	0.62^3^
Significant	4 (8.3)	3 (11.5)	1 (4.8)	
ICU Discharge				
Severe	15 (32.6)	5 (19.2)	10 (50.0)	0.04^2^
Significant	20 (43.5)	12 (46.2)	8 (40.0)	
Normal	11 (24.0)	9 (34.6)	2 (10.0)	
*MMS (ICU Discharge)*				
Sitting on the edge of the bed	2 (4.2)	1 (3.8)	1 (4.8)	0.20^4^
Hoist to a chair	11 (22.9)	3 (11.5)	8 (38.1)	
Standing	18 (37.5)	13 (50.0)	5 (23.8)	
Step transfer to chair	11 (22.9)	6 (23.1)	4 (19.0)	
Mobilise < 30 Meters	3 (6.3)	2 (7.7)	1 (4.8)	

*CPAP* continuous positive airway pressure, *MMS* Mobilization of the Myofascial System, *MRC* Medical Research Council, *TT* tracheostomy tube

*Presented as *n* (%) unless otherwise indicated

**Based on MRC

^1^ T-test

^2^Chi-Squared

^3^Fishers

^4^Likelihood Ratio

Impaired airway protection on swallow during nasendoscopy (as defined by a PAS score of 3 or higher) was observed in 21 (43.8%). At the time of nasendoscopy, impaired airway protection was significantly associated with an inability to swallow fluids and solids safely for 29 (60.4%; *p* = 0.006) and 38 (79.2%; *p* = 0.001) patients respectively. Those with and without normal airway protection did not differ significantly in regards to demographic and baseline variables except for BMI (*p* = 0.04) and asthma (*p* = 0.003). Those with normal airway protection had greater BMI and very few patients in our sample had underlying asthma. Impaired airway protection (as determined on PAS) was associated with longer total duration of artificial airway (*p* = 0.008), longer tracheostomy tube duration (*p* = 0.007), and multiple intubations (*p* = 0.006). Airway protection impairment was also significantly different in those with greater critical care weakness upon ICU discharge (Table 2).

### Supraglottic and Glottic Findings

We observed marked airway oedema with 32 (66.7%) patients scoring 3 or above on at least one revised Patterson oedema Scale component. Oedema scores ≥ 3 were present in at least one anatomical area for 29 (60.4%) patients in region 1, and for 22 (45.8%) in region 2. When considering the individual glottic components (region 1) and airway protection, lower oedema scores favored normal airway protection for false vocal fold movement/oedema (*p* = 0.05). For supraglottic components (region 2), normal airway protection was associated with lower oedema scores for the valleculae (*p* = 0.01). Those with normal airway protection had significantly better management of their secretions (*p* =  < 0.001). Individual oedema scale components and secretion scores are presented in Table 3. Following initial nasendoscopy, 19 (40%) of patients were able to receive oral fluids and 10 (21%) were able to receive modified texture solid food. Normal airway protection was significantly associated with the ability to consume oral fluids (*p* = 0.003) and food (*p* = 0.006). Following our exploratory regression (Table 4), an independent predictor for impaired airway protection was artificial airway duration (OR 1.05 [95%CI 1.01–1.09], *p* = 0.02). One patient presented with a vocal cord praxis which recovered within 30 days and didn’t preclude decannulation, another presented with a vocal cord palsy and required targeted SLT rehab before the tracheostomy was also removed successfully. No granuloma or stenosis were identified.

**Table 3:** Upper airway characteristics according to scale across the sample

Scale*	All patients (*N* = 48)		Airway protection		*p*-value
			Normal (*n* = 26)	Impaired (*n* = 21)	
Patterson Oedema Scale (%)					
**Region 1: Glottis**					
Component	Score				
Aryepiglottic folds	1	7 (14.6)	6 (23.1)	1 (4.8)	0.13^4^
	2	24 (50.0)	13 (50.0)	11 (52.4)	
	3	11 (22.9)	6 (23.1)	5 (23.8)	
	4	5 (10.4)	1 (3.8)	4 (19.0)	
Arytenoid	1	3 (6.3)	1 (3.8)	2 (9.5)	0.36^4^
	2	18 (37.5)	11 (42.3)	6 (28.6)	
	3	18 (37.5)	11 (42.3)	7 (33.3)	
	4	9 (18.8)	3 (11.5)	6 (28.6)	
False vocal folds	1	6 (12.5)	5 (19.2)	1 (4.8)	0.05^4^
	2	34 (70.8)	20 (76.9)	14 (66.7)	
	3	6 (12.5)	1 (3.8)	5 (23.8)	
	4	1 (2.1)	0 (0)	1 (4.8)	
True vocal folds	1	15 (31.3)	12 (46.2)	3 (14.3)	0.07^4^
	2	25 (52.1)	11 (42.3)	14 (66.7)	
	3	6 (12.5)	3 (11.5)	3 (14.3)	
	4	1 (2.1)	0 (0)	1 (4.8)	
**Region 2: Supraglottis**					
Epiglottis	1	9 (18.8)	5 (19.2)	4 (19.0)	0.48^4^
	2	27 (56.3)	16 (61.5)	10 (47.6)	
	3	11 (22.9)	5 (19.2)	6 (28.6)	
	4	1 (2.1)	0 (0)	1 (4.8)	
Pharyngoepiglottic folds	1	5 (10.4)	4 (15.4)	1 (4.8)	0.07^4^
	2	25 (52.1)	15 (57.7)	9 (42.9)	
	3	15 (31.3)	7 (26.9)	8 (38.1)	
	4	3 (6.3)	0 (0)	3 (14.3)	
Pyriform sinus	1	5 (10.4)	3 (11.5)	2 (9.5)	0.17^4^
	2	33 (68.8)	20 (76.9)	12 (57.1)	
	3	8 (16.7)	3 (11.5)	5 (23.8)	
	4	2 (4.2)	0 (0)	2 (9.5)	
Vallecula	1	18 (37.5)	14 (53.8)	4 (19.0)	0.01^4^
	2	26 (54.2)	12 (46.2)	13 (61.9)	
	3	2 (4.2)	0 (0)	2 (9.5)	
	4	2 (4.2)	0 (0)	2 (9.5)	
Secretion scale (%)	1	27 (56.3)	23 (88.5)	4 (19.0)	< 0.001^4^
	2	5 (10.4)	2 (7.7)	3 (14.3)	
	3	15 (31.3)	1 (3.8)	14 (66.7)	

*All numbers represent *n* (%) unless otherwise indicated

**Table 4:** Independent predictors of Impaired airway protection

Variable	*p*-value	Odds Ratio	95% CI
Age	0.09	1.07	0.99 to 1.16
Male	0.83	0.85	0.19 to 3.84
Critical care acquired weakness^a^	0.17	3.50	0.58 to 21.08
False vocal fold oedema^b^	0.81	0.72	0.49 to 10.67
Artificial airway duration^c^	0.02	1.05	1.01 to 1.09

^a^Severe and very severe weakness

^b^At least moderate oedema

^c^Duration in days for both endotracheal and tracheostomy tubes

### Outcomes Following Rehabilitation

Mean (SD) length of stay in the ICU was 43.7 (21.6) days. Those with impaired airway protection had significantly longer mean (SD) length of ICU stay (days) when compared to those with normal airway protection: (53.6 (27.2) versus 36.6 (12.6); *p* = 0.02). Upon discharge from ICU, 19 (39.6%) had severe to very severe ICU acquired weakness with 3 (6.3%) able to mobilize < 30 m. Critical care acquired weakness on ICU discharge was significantly different between groups with impaired airway protection (*p* = 0.04); impaired airway protection was also an independent predictor for tracheostomy tube duration (Table 5, *p* = 0.02, Beta 0.38, 95% CI 2.36 to 27.16). Mean (SD) ward length of stay was 27.7 (19.2) days with an overall hospitalization duration of 71.4 (30.4) days. At hospital discharge, the majority 31(64.6%) tolerated regular fluids and solids (with only one patient remaining nil by mouth). There were no significant differences between those with and without normal airway protection and physical rehabilitation outcomes at discharge. Furthermore, overall hospitalization durations were not significantly different between those with and without normal airway protection. Details regarding physical rehabilitation outcomes are available in Table 6. No patients were readmitted to hospital with swallowing or aspiration events following discharge.

**Table 5:** Independent predictors of tracheostomy duration

Variable	*p*-value	Beta	95% CI
Age	0.33	− 0.16	− 0.86 to 0.30
Male	0.79	0.04	− 10.39 to 13.53
Critical care acquired weakness^a^	0.74	0.05	− 10.96 to 15.38
False vocal fold oedema^b^	0.16	0.24	− 5.15 to 31.08
Impaired airway protection^c^	0.02	0.38	2.36 to 27.16

^a^Severe and very severe weakness

^b^At least moderate oedema

^c^Defined as a PAS >  = 3

**Table 6:** Swallowing and length of stay outcomes

Outcome*		All patients (*N* = 48)	Airway protection			*p-*value
			Normal (*n* = 26)	Impaired (*n* = 21)		
Swallowing recommendations						
*Initial assessment *(%)						
Diet	None	38 (79.2)	17 (65.4)	21 (100.0)		0.006^4^
	Level 3	6 (12.5)	5 (19.2)	0 (0)		
	Level 4	2 (4.2)	2 (7.7)	0 (0)		
	Level 6	2 (4.2)	2 (7.7)	0 (0)		
Fluids	None	29 (60.4)	11 (42.3)	17.0 (85.7)		0.003^4^
	Level 0	13 (27.1)	11 (42.3)	1 (4.8)		
	Practice swallows	6 (12.5)	4 (15.4)	2 (9.5)		
*Discharge *(%)						
Diet	None	1 (2.1)	0 (0.0)	1 (4.8)		0.27^4^
	Level 4	1 (2.1)	1 (3.8)	0 (0)		
	Level 6	2 (4.2)	1 (3.8)	0 (0)		
	Level 7	43 (89.6)	24 (92.3)	19 (90.5)		
Fluids	None	1 (2.1)	0 (0.0)	1 (4.8)		0.25^4^
	Level 0	45 (93.8)	25 (96.2)	19 (90.5)		
	Level 1	1 (2.1)	1 (3.8)	0 (0.0)		
Lengths of stay**						
*ITU* (%)						
Mean (SD)		43.7 (21.6)	36.6 (12.6)		53.6 (27.2)	0.02^1^
Median (IQR)		40.0 (23.0)	37.0 (15.0)		47.0 (30.0)	
*Ward* (%)						
Mean (SD)		27.7 (19.2)	26.0 (19.7)		30.8 (18.8)	0.41^1^
Median (IQR)		24.0 (17.0)	21.5 (21.0)		29.0 (17.0)	
*Total hospitalization* (%)						
Mean (SD)		71.4 (30.4)	62.6 (26.6)		84.4 (31.4)	0.07^1^
Median (IQR)		66.0 (31.0)	60.0 (39.0)		71.0 (49.0)	

Level 0 = Thin fluids, Level 1 = Slight thick fluids, Level 3 = Moderately thick liquidized diet, Level 4 = Pureed diet, Level 6 = Soft and bite sized food, Level 7 = Regular food

*ITU* Intensive Treatment Unit

*Presented as *n* (%) unless otherwise indicated

**Reported in Days

^1^ T-test

^2^Chi-Squared

^3^Fishers

^4^Likelihood Ratio

## Discussion

Since the emergence of COVID-19, publications exploring tracheostomy pathways [18, 19] have enhanced understanding of how tracheostomy may improve outcomes. Few, however, have detailed decannulation rates or provided operational guidance on how to optimise outcomes, like swallowing, while informing acute decision-making. Within our data, at initial assessment over two-thirds presented with an oedema score of 3 or above with impaired airway protection evident in nearly half, lower oedema scores favored normal airway protection. Furthermore, we explored predictors for impaired airway protection and tracheostomy tube duration. Our collective findings not only align with presenting otolaryngological manifestations of COVID-19 (e.g. pharyngeal erythema) [20] and laryngeal pathologies following intubation in general [21], they also suggest that inclusion of a functional, multi-disciplinary laryngeal assessment is beneficial. Although decannulation timing may be influenced by many variables, our assessment approach enables clinical teams to make practical risk stratification for decannulation with findings pertinent to ENT surgeons, SLT’s and Intensivist decision-making.

Patients with impaired airway protection had significantly more complex respiratory recovery specifically longer durations of artificial airways (both endotracheal and tracheostomy tubes) and more frequent intubations. In addition, the majority required downsizing or fenestrated tubes. Although our analyses were exploratory, we suggest impaired airway protection may be an independent predictor of tracheostomy duration. While this aligns with other studies on critically ill patients without COVID-19 [22], this is the first exploration for this novel population. As a result, using objective laryngeal measurements such as the revised Patterson Oedema scale [12] will afford objective characterization while facilitating a systematic method to monitor change. This enables efficient treatment, standardised recovery monitoring, and streamlining of decannulation processes and resource allocation. Doing so within the ICU is particularly prudent where bed availability is at a premium, particularly during this pandemic.

A common finding in survivors of critical illness is ICUAW [23]. The majority of our patients (~ 80%) had a CFS < 3, indicating that they were very fit, fit or functioning well prior to admission. Despite this, all had significant or severe ICUAW at rehabilitation commencement. Furthermore, patients with ongoing airway protection issues at ICU discharge were significantly more likely to have persistent ICUAW weakness. In general, physical rehabilitation focuses on extremities and outcomes related to activities of daily living (e.g., ambulation) [24]. In contrast, the impact of weakness on swallowing particularly following artificial airway use is extremely limited [25]. While investigating the relationship between ICUAW and dysphagia was not the objective of our study, given the limited evidence in this area, routine screening for the presence of ICUAW using the MRC score, particularly on waking from sedation, may be useful. Not only may it prompt nasendoscopic airway assessment, it may also highlight those who would most benefit from SLT assessment and rehabilitation. Furthermore, future mechanistic studies of swallowing in this population would inform bespoke rehabilitation approaches.

This was a quality assurance audit, undertaken during the third COVID-19 surge in the UK and as a result, our study had limitations and should be considered through the lens of the following design caveats. Our small sample size without a control group limits generalizability and does not elucidate the potential differences between this populations as compared to those with critical illness without COVID-19. However, it is pertinent to couch this methodological limitation in line with other pandemic publications without control groups, which have contributed fundamental learning to this novel and emergent pathophysiology and clinical presentation [26]. In addition, our small sample size lent itself to analyses primarily focused on associations. In the future, conducting multi-variate regressions with statistically informed models and multiple outcome variables would be useful to develop predictive risk profiles and support practice. As with most clinical research on novel diagnostic groups, the impairment scales used herein have not been validated on this population specifically, however, given the ability of the tools to describe the laryngeal pathology, we suggest this may be clinically valuable to teams moving forwards whilst reliability and validity tests are undertaken. As this was a clinically based team, there was no blinded scoring of the laryngeal assessment increasing risk of confirmation bias. Regardless, our findings offer the first systematic approach to functional airway assessment following tracheostomy and severe COVID-19, offering unique information to clinical teams managing this challenging clinical presentation of laryngeal compromise.

## Conclusion

Our findings highlight the functional relationships between the anatomy and physiology of the larynx and cumulative outcomes following artificial airway insertion. In our institution, patients who required tracheostomy following COVID-19 presented with impaired airway protection and marked airway oedema in at least one laryngeal area. Impaired airway protection was associated with longer total duration of artificial airway, longer tracheostomy tube duration, and multiple intubations. We suggest proactive assessment, standardised scoring, and patient risk stratification to enable the clinical team to create collaborative and effective decannulation plans.
